# Silicon Effect on Conductive Behavior in Rubber Recycled Composites

**DOI:** 10.3390/polym18010137

**Published:** 2026-01-02

**Authors:** Marc Marín-Genescà, Ramon Mujal Rosas, Jordi García Amorós, Lluis Massagues, Xavier Colom

**Affiliations:** 1 Mechanical Engineering Department, Escola Tècnica Superior d’Enginyeria Química, Rovira i Virgili University, 43007 Tarragona, Spain; 2 Electrical Engineering Department, Escola d’Enginyeria de Terrassa, Technical University of Catalonia, 08222 Terrassa, Spain; 3 Electrical Engineering Department, Escola Tècnica Superior d’Enginyeria, Rovira i Virgili University, 43007 Tarragona, Spain; jordi.garcia-amoros@urv.cat (J.G.A.); lluis.massagues@urv.cat (L.M.); 4 Chemical Engineering Department, Escola d’Enginyeria de Terrassa, Technical University of Catalonia, 08222 Terrassa, Spain; xavier.colom@upc.edu

**Keywords:** devulcanization, rubber recycling composites, dielectric behavior

## Abstract

In the present research, the structure and thermal–dielectric behavior of Styrene Butadiene Rubber (SBR) and of the SBR/EPDMd composite with SiO_2_ with different compositions and concentrations of EPDMd are analyzed. In this sense, interesting behaviors are observed for the DC-AC regime of the conductive behavior of the material; therefore, a very marked DC and AC regime is observed in the conductivities, showing a different dielectric behavior at low and high frequencies. On the other hand, peak relaxations due to polarization phenomena are observed in terms of the imaginary modulus. Conductively, SiO_2_ does not produce significant or relevant changes, but it does produce changes in the permittivity and the electrical modulus, so it is concluded that the impact of the incorporation of SiO_2_ in these compounds affects energy storage (permittivity and modulus) in these types of compounds. Compared with compounds without silica (insights—no SiO_2_), it is observed that SiO_2_ maintains a similar operating regime to the initial one (SBR and SBR + EPDMd + SiO_2_) without SiO_2_ dielectric changes occurring, so silica presence modifies the dielectric behavior, reducing polarization effects, as can be seen in the dielectric results. Conductively, SiO_2_ produces more insulating compounds, that is, less conductive; this property can make it interesting as electrical insulation.

## 1. Introduction

The recycling of rubbers such as the Ethylene–Propylene–Diene Monomer (EPDM) [1,2,3] has been one of the goals of researchers who have focused on the study of the behavior of rubbers used in industry, such as EPDM. On the other hand, Styrene Butadiene Rubber (SBR) [4,5] is a synthetic elastomer obtained through the polymerization of a mixture of monomers: styrene and butadiene. It is a rubber with the largest volume of world production. Its main application is in the manufacture of tires, and it is the rubber with the largest volume of consumption. EPDM is widely used in industrial and construction applications, such as sealants, joints, and roof coverings, thanks to its excellent resistance. However, the mass production and intensive use of EPDM generate waste that can have significant environmental impacts if not managed properly. In this context, EPDM recycling becomes key: it allows us to reduce our dependence on petrochemical resources, reduce the accumulation of waste in landfills, and reduce the emission of polluting gases derived from incineration.

### 1.1. Devulcanization of EPDM

EPDM, like other elastomers, is vulcanized to improve its elasticity and mechanical strength. To recycle it effectively, it is necessary to partially reverse this process through devulcanization, which breaks the polymer’s cross-links. There are several prominent methods [1,2,3]:

1. Microwave (MW) devulcanization [6,7,8]: this method applies microwave energy to heat the material from the inside, favoring the selective breaking of sulfur bonds. The main advantages include less degradation of the polymer and a relatively fast and energy-efficient process. MW devulcanization allows for obtaining recyclable EPDM with properties like those of the original material.
2. Chemical devulcanization [9,10]: this method uses specific chemical agents that break sulfur bonds and other cross-links. Depending on the agent used, a high degree of devulcanization can be achieved, but conditions must be precisely controlled to avoid the degradation of the polymer main chain and loss of mechanical and elastic properties.

### 1.2. Silica Incorporation and Effects on Properties

Once devulcanized EPDM is recovered, it is often reinforced with fillers such as silica to improve the properties of the compounds. Silica plays a key role in the following aspects [11,12,13]:

Mechanical properties: The addition of silica increases the stiffness, abrasion resistance, and durability of the compound. This is due to the interaction between the silica surface and the polymer chains, which reinforces the matrix and better distributes the applied stresses. Thus, the final products maintain a good elasticity and fatigue resistance, even after recycling.

Electrical properties: Silica has a stabilizing effect, as it is a dielectric material and helps reduce the conductivity of the compound. This is especially important in applications such as cables and electrical insulation, where a low level of conductivity and good discharge resistance are crucial.

Silica-modified rubbers, such as SBR and EPDM composites, are widely used not only for mechanical reinforcement but also for tuning dielectric properties, making them ideal systems to study interfacial effects, cure kinetics, and polymer–filler interactions. The incorporation of silica (SiO_2_) enhances thermal stability, improves ionic mobility control, and increases interfacial polarization, which can be monitored through techniques such as Dynamic Electrical Analysis (DEA). Previous studies have shown that SBR/SiO_2_ composites exhibit a higher permittivity and lower dielectric loss compared to unfilled SBR, while EPDM/SiO_2_ systems demonstrate an improved dielectric stability and thermal resistance [14,15,16,17,18]. Although hybrid blends of SBR and EPDM with silica are less explored, they offer the potential to optimize both mechanical and dielectric performance, making silica an effective choice for investigating the thermodynamic compatibility of polymer phases and filler dispersion in multi-component rubber composites.

In summary, the recycling of EPDM through devulcanization (mechanical or chemical) and the subsequent incorporation of silica enable the production of compounds with improved mechanical and electrical properties, maximizing the use of materials and contributing to environmental sustainability. This combination of techniques is essential for the development of a more ecological and efficient rubber industry.

The objective of this article is the study and analysis of the physical properties, especially dielectric, in the presence of silica of SBR samples; in these samples, the presence of silica can be useful to make the component more resistant at a mechanical level [19], but it is also necessary to study whether this additive can influence dielectric behavior, and what effect it may have. In addition, it is necessary to determine if this incorporation of silica makes samples more insulating from a dielectric point of view. So, as a summary, the purpose of the article is to provide the complete dielectric behavior of SBR + EPDMd + SiO_2_ composites, to compare the addition of SiO_2_ to the SBR + EPDMd matrix, and finally to analyze possible dielectric applications.

## 2. Methodology

### 2.1. Materials and Methods

SBR stabilized with butylated hydroxytoluene antioxidant (BHT) (0.375%), bound styrene 24%, stearic acid 5.5% and Mooney Viscosity (ML + 1@100 °C) 52 was supplied by VIGAR Rubí Barcelona (Spain). Micro/nanoparticles of SiO_2_ (83%): the particles have a size below 150 µm and a specific surface area of 180 m^2^/g, indicating a very fine morphology. Waste EPDM of commercial, industrial, and residential roofing origin was supplied by Firestone Building Products Terrassa (Spain). The additives and curing agents used for curing, vulcanization accelerators (TBBS-N-tert-butyl-2-benzothiazole sulfonamide, TMTD—tetramethyl thiuram disulfide), carbon black N550 (ASTM D1765), stearic acid, zinc oxide, and sulfur with technical purity were supplied by VIGAR Rubí Barcelona (Spain).

### 2.2. Devulcanization of EPDM

The devulcanization process of EPDM by combined thermomechanical/microwave procedures has been carried out according to the procedure already tested and published by our group in a previous paper [20,21]. The thermo-mechanical treatment is carried out in a Brabender plastograph (Duisburg, Germany) at 80 °C and 80 rpm, including the addition of 2 phr of benzoyl peroxide (devulcanizing agent), which helps the thermomechanical devulcanization. The microwave irradiation takes place in a prototype microwave oven with motorized stirring, adapted in our laboratory. The microwave devulcanization process was performed, setting the magnetron power to 700 W, 80 rpm for the stirrer, and 3 min of MW exposure. The resulting material after this process has been named EPDMd.

### 2.3. Samples Preparation

**Figure 1:**
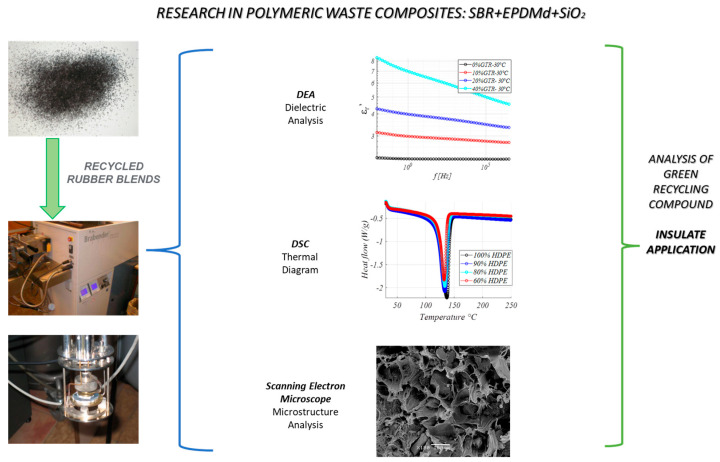
Summary of methods used in the present research.

The formulations of elastomeric compounds are shown in Table 1. The mixing process consisted of three steps. In the first step, a preliminary plasticization of the SBR fresh elastomer take place for 2 min afterwards, followed by 4 min of mixing with EPDMd, 30 phr of carbon black, and 30 phr of SiO_2_, and finally 2 min of mixing the blend with the sulfur curing system (zinc oxide 5.0 phr; stearic acid 3.0 phr; N-tert-butyl-2-benzothiazolesulfenamide (TBBS) 1.0 phr; Tetramethylthiuram disulfide (TMTD) 0.25 phr; sulfur 2.0 phr). The rubber ingredients were mixed in a Plasticorder internal mixer (Brabender GmbH & Co. KG, Duisburg, Germany), as is seen in Figure 1, at 80 °C and 80 rpm. The order of appearance for the components in Table 1 (left to right) also reflects the order of mixing. The devulcanized EPDM content in the samples was 0, 20, and 40 parts per hundred of rubber (phr).

The resultant elastomeric compounds obtained are taken to the two-roll mill Collin Teach-line, which is used to mix the two batches required to make a single plate. Afterwards, the mold and the Collin P 200 E hot plate press are prepared at 160 °C and 200 bar. These conditions correspond to the optimum cure time (t90) determined by the vulcanization characteristics of vulcanizable rubber compounds, as per ASTM D2084, using an Oscillating Disc Rheometer R100 (Monsanto, Akron, OH, USA) at 162 ± 1 °C. After this time, the mold is removed with Kevlar gloves and quickly taken to cool. Finally, the mold is separated from the part, which is now ready for the extraction of test samples with a J.BOT Instruments S.A. (Barcelona, Spain) test cutter, which has been minted in the form of a dumbbell test according to the specifications of the ASTM-D-412-98.

TGA was performed on a Perkin Elmer TGA/SDTA 8000 apparatus (USA). An SBR/GTR composite weighing approximately 10 mg was placed in a corundum dish. The measurement was conducted in the temperature range 30–800 °C and under oxidant atmosphere (30 mL/min), at a heating rate of 20 °C/min. The obtained results are the average of three measurements per sample.

The morphology of SBR/EPDMd fracture surfaces created by the tensile test of the composites at a speed of 500 mm/min was observed with a JEOL 5610 scanning electron microscope (Japan). Before observation, the samples were covered with a fine gold–palladium layer to increase their conductivity in a vacuum chamber.

A dielectric test (DEA) has been performed using the Dynamic Electric Analysis Test from Novocontrol. This test utilizes electrodes and performs a dielectric test over a frequency range of 0.01 Hz to 3 × 10^6^ Hz. Accuracy is not constant as a function of frequency; below 0.1 Hz, the errors are up to 5%, and in the 0.1 Hz–1 kHz range the error can be approximately 1%.

## 3. Results and Discussion

### 3.1. Thermal Behavior Analysis

**Figure 2:**
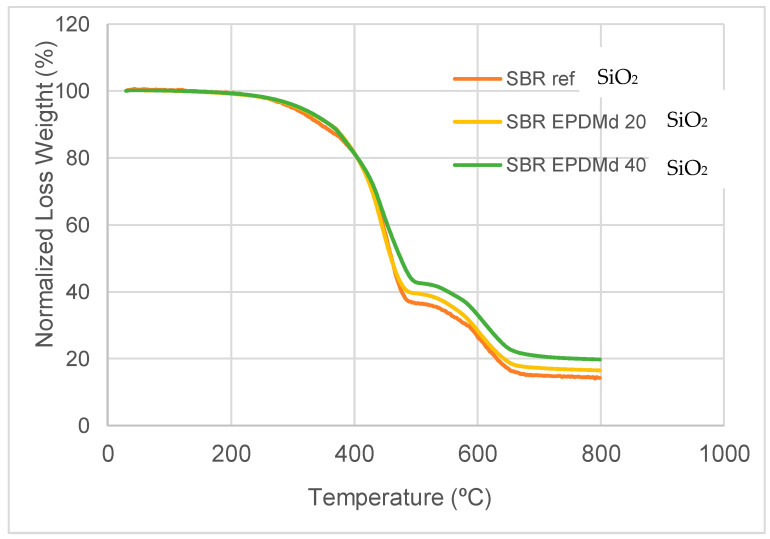
Thermogravimetric test, from 30 °C to 800 °C. Y axis, Normalized Loss Weight (in Percent).

The TGA results provide evidence of the thermodynamic compatibility among SBR, EPDM, and SiO_2_. A single-step thermal degradation and an increase in onset temperature upon the addition of silica suggest a good miscibility of the polymer phases and effective filler dispersion. Regarding thermal behavior, we see, in Figure 2, how it has great similarities in the range from 30 to 450 °C, but from this point on the effect of SiO_2_ is noticeable, with an increase in the Loss Weight (Y axis) for compounds in the presence of SiO_2_.

**Figure 3:**
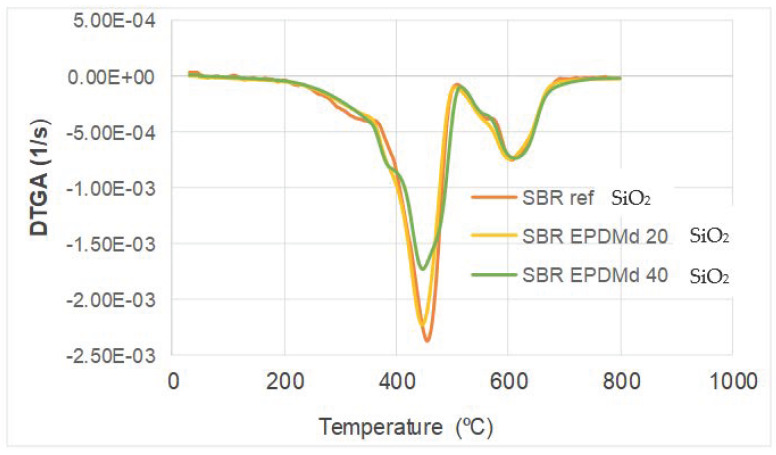
Differential TGA. Thermogravimetric analysis mass loss curves.

On the other hand, in Figure 3, a DTGA is shown; in this we observe a thermal peak at 430 °C where the most important mass loss occurs during the TGA thermal process for the SBR sample only. At this point, we can see great differences with the presence of silica, since for the maximum presence of SiO_2_ the reduction in the thermal peak is very relevant.

In summary, the thermal behavior is very similar to Styrene Butadiene Rubber with SBR/EPDMd [15], but the presence of silicon affects this behavior, reducing the peak in this temperature point (430 °C), which is consistent with the previously published works [22,23].

### 3.2. Structure

**Figure 4:**
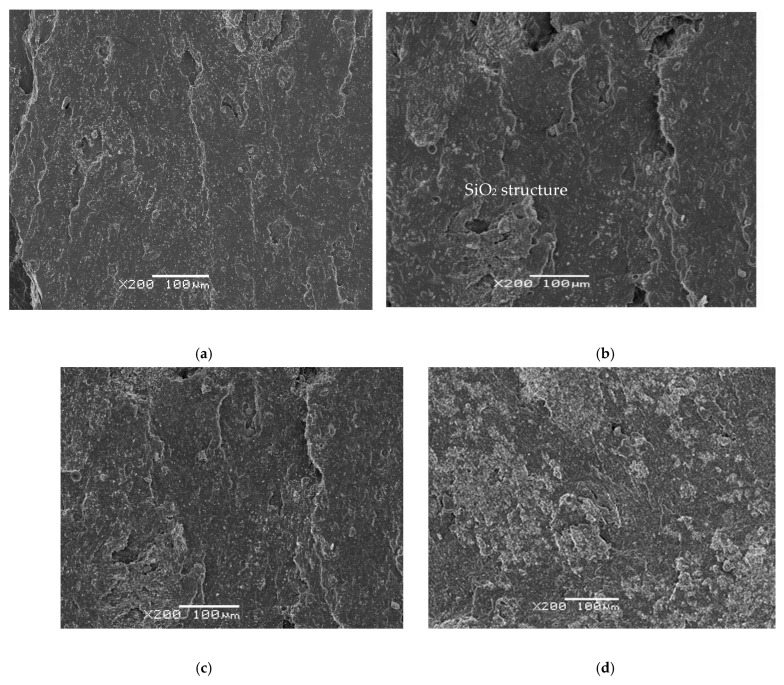
(**a**) SBR100 (magnification × 100) and (**b**) SBR 100 (magnification × 1500); (**c**) SBR 20EPDMd augments; (**d**) SBR40 EPDMd (magnification × 100).

In the structural analysis (Figure 4a,b) of the SBR compound with SiO_2_, a homogeneous structure is observed with the SBR samples at 100 to 200 (a) and at 1500 (b) magnifications. It is with the incorporation of the SBR/EPDMd samples with silica (c) that we begin to perceive disconnected silica particles as well as the presence of cracks; also, with the growth of the proportion of silica (40%) in Figure 4d, this tendency increases definitively.

Despite the presence of silica particles and certain discontinuities in the images, due to the irregular incorporation of SBR with EPDMd, a homogeneity is observed in the analyzed samples, so we deduce that these samples have been developed with the correct procedures and methods, avoiding the agglomeration of the additive in the samples. The discontinuities in the analyzed composites grow with the incorporation of silica; other structural studies show the presence of silica [24,25,26].

### 3.3. Dielectric Characteristics

#### 3.3.1. Real Permittivity: ε′, Dielectric Constant

The spectrum of dielectric permittivity covers a wide range of frequencies. The real and imaginary parts of the permittivity are shown, and several processes are represented: ionic and dipolar relaxations, and atomic and electronic resonances at high energies [17,27,28,29]. The permittivity is determined by the tendency of a material to polarize before the application of an electric field. It is directly related to electrical susceptibility. SiO_2_ has excellent dielectric properties, a very high resistivity of ~10^18^ cm, and a large band gap of ~9 eV. Silica generally served as the dielectric material for silicon-based microelectronic applications for decades, with good resistance, electrical insulation, and thermal stability.

**Figure 5:**
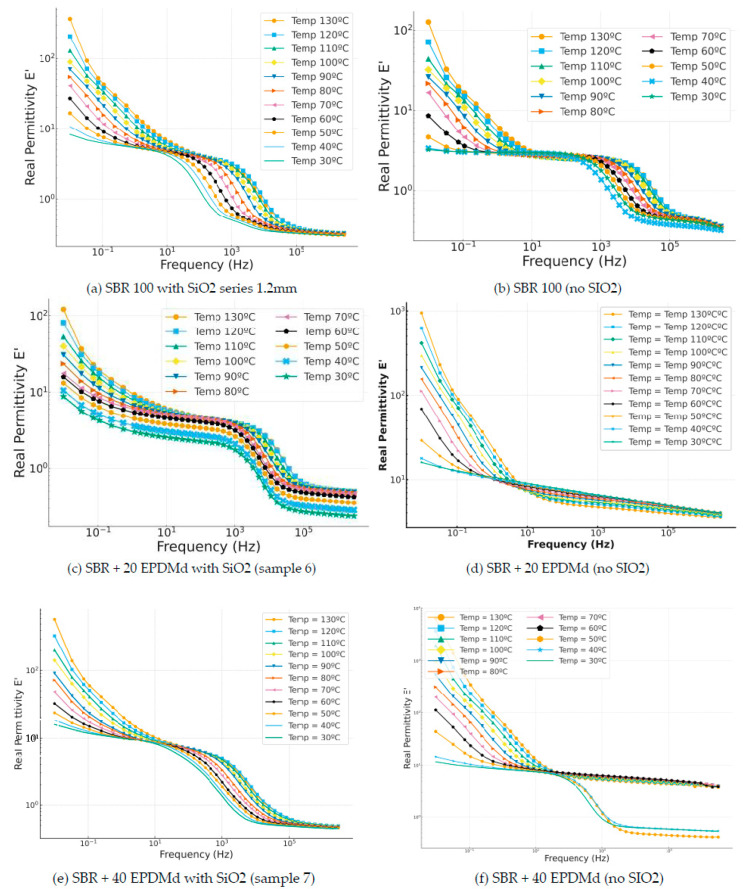
Real permittivity part (ε′) versus frequency (Hz) in temperature dependence (°C) of Styrene Butadiene Rubber (SBR) and SBR-EPDMd composites, with SiO_2_ and no SiO_2_ addition: (**a**) SBR100 + SiO_2_; (**b**) SBR100; (**c**) SBR100 + 20EPDMd + SiO_2_; (**d**) SBR + 20%EPDMd; (**e**) SBR100 + 20EPDMd + SiO_2_; (**f**) SBR + 40%EPDMd.

In this case, in Figure 5, the incorporation of silica (Figure 5c) produces a reduction in the dielectric constant of 0.3 compared to the compound without silica (Figure 5d), in which we can observe how the dielectric constant is reduced linearly, with a less relevant reduction than with sample Figure 5c. In the SBR 40 EPDMd samples with SiO_2_ (Figure 5e), homogeneous behavior is observed as a function of temperature in the frequency range, but in Figure 5f, the SBR 40 EPDMd sample without silica, we see two differentiated behaviors (40–30 °C) and another for temperatures higher than 50 °C. It could be said that SiO_2_ produces a masking of the dielectric effects that we have without SiO_2_; that is, SiO_2_ is a dielectric material, but its dielectric characteristics allow the incorporation of SiO_2_ into SBR, showing a dielectric behavior like that of pure SBR. This is because SiO_2_ is itself an insulating material, so it does not significantly change the electrical properties of the composite.

#### 3.3.2. Imaginary Permittivities: ε″, Loss Factor

**Figure 6:**
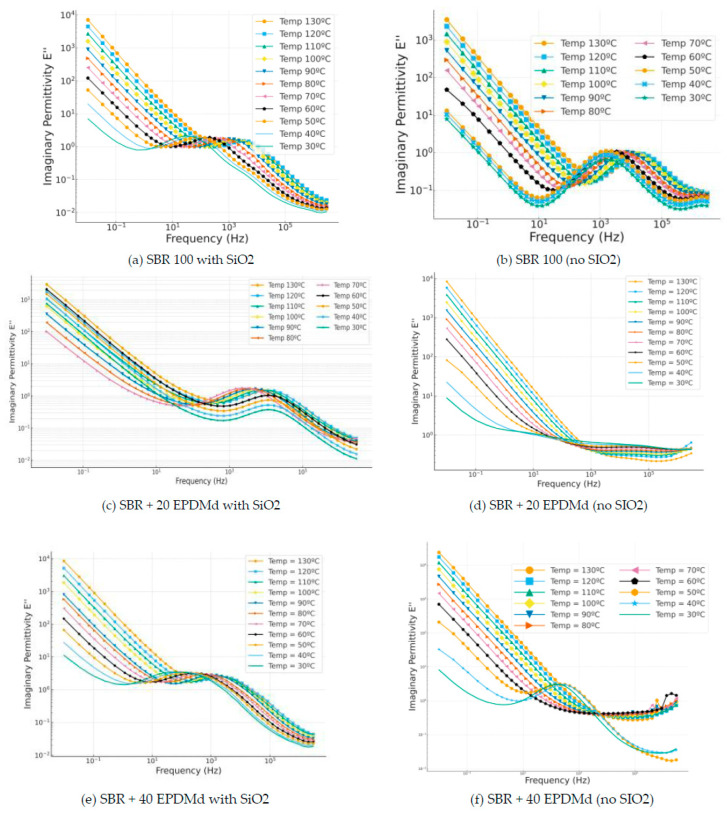
Imaginary permittivity part (ε”) versus frequency (Hz) in temperature dependence (°C) of Styrene Butadiene Rubber (SBR) and SBR-EPDMd composites, with SiO_2_ and no SiO_2_ addition: (**a**) SBR 100 with SiO_2_; (**b**) SBR 100; (**c**) SBR 100 + 20EPDMd with SiO_2_; (**d**) SBR + 20%EPDMd; (**e**) SBR 100 + 20EPDMd with SiO_2_; (**f**) SBR + 40%EPDMd.

In Figure 6a,c,e, we see how the incorporation of SiO_2_ shows very similar behavior in the three cases, portraying a masking effect of Maxwell–Wagner–Sillars (MWS)-type relaxations [30,31,32,33] in the three cases of SBR and compounds with 20% and 40% EPDMd. On the other hand, in the samples without silica, the MWS relaxation largely disappears, which is observed in the samples with silica.

#### 3.3.3. Dielectric Modulus M′

**Figure 7:**
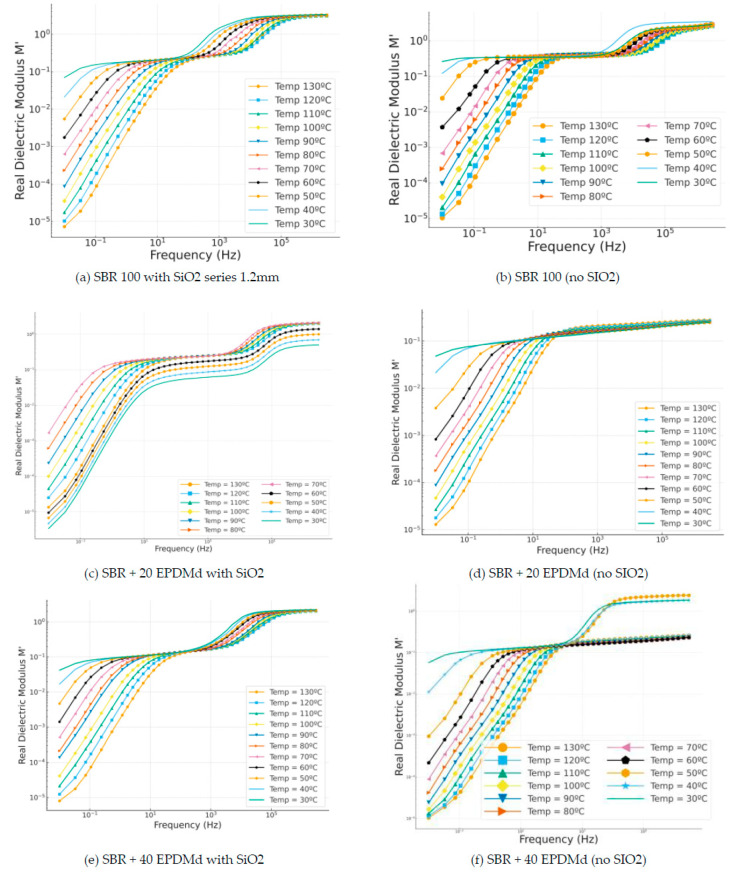
Real modulus part (M′) versus frequency (Hz) in temperature dependence (°C) of Styrene Butadiene Rubber (SBR) and SBR-EPDMd composites, with SiO_2_ and no SiO_2_ addition: (**a**) SBR 100 with SiO_2_; (**b**) SBR 100; (**c**) SBR 100 + 20EPDMd with SiO_2_; (**d**) SBR + 20%EPDMd; (**e**) SBR 100 + 20EPDMd with SiO_2_; (**f**) SBR + 40%EPDMd.

In Figure 7a,b, we have similar behaviors in which a reflection or peak is seen at the frequency 10^4^ Hz, a frequency coinciding with the reflection of dipolar structures; in this sense, this dielectric reflection, called dipolar impedance spectroscopy (IONS—DIPOLS), from 10^4^ Hz is also observed in those that incorporate silica (Figure 7c). However, in the same compounds (Figure 7d), this dipolar reflection (MWS) does not appear. Finally, the dielectric behavior in samples Figure 7e,f is very similar, presenting differentiated dielectric regimes depending on the temperature. 

#### 3.3.4. Dielectric Modulus M″

The dielectric modulus formula is widely used to analyze the dielectric behavior of insulating materials, and is expressed according to the following Equations (1) and (2):

(1) $$M^{*}\;=\;M^{\prime }\;+\;jM\prime \prime \;=\;jw\varepsilon_{0}Z^{*}\;=\;jw\varepsilon_{0}Z^{\prime }\;-\;jw\varepsilon_{0}Z\prime \prime$$

(2) $$M^{*}\;=\;1/\varepsilon^{*}\;=\;1/(\varepsilon^{\prime }\;-\;j\;\varepsilon \prime \prime )\;=\;M^{\prime }\;+\;j\;M\prime \prime \;=\;[\varepsilon^{\prime }/({\varepsilon^{\prime }}^{2}\;+\;{\varepsilon \prime \prime }^{2})]\;+\;j\;[\varepsilon \prime \prime /({\varepsilon^{\prime }}^{2}\;+\;{\varepsilon \prime \prime }^{2})]$$

**Figure 8:**
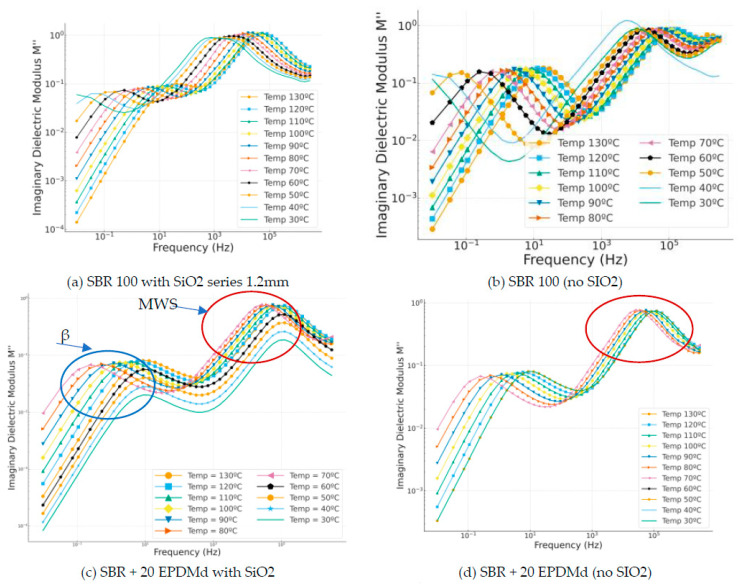

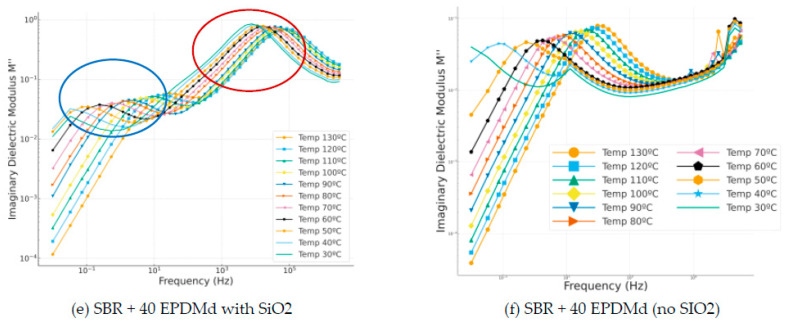
Imaginary modulus part (M″) versus frequency (Hz) in temperature dependence (°C) of Styrene Butadiene Rubber (SBR) and SBR-EPDMd composites, with SiO_2_ and no SiO_2_ addition: (**a**) SBR 100 with SiO_2_; (**b**) SBR 100; (**c**) SBR 100 + 20EPDMd with SiO_2_; (**d**) SBR + 20%EPDMd; (**e**) SBR 100 + 20EPDMd with SiO_2_; (**f**) SBR + 40%EPDMd.

The imaginary modulus is a very interesting reference for studying dielectric reflections, which are peaks that occur as a function of frequency. These peaks help us to study the material. In the SBR samples with SiO_2_ (Figure 8a,c,e), two dielectric relaxation peaks are very clearly observed: 100 for beta relaxation and 10^4^ for MWS relaxation present in the sample due to this incorporated silica. On the other hand, in the samples without silica (Figure 8d,f), the first relaxation peak (beta) is observed, but the second peak (MWS) is not perceived in the frequency range studied.

#### 3.3.5. Conductivity

**Figure 9:**
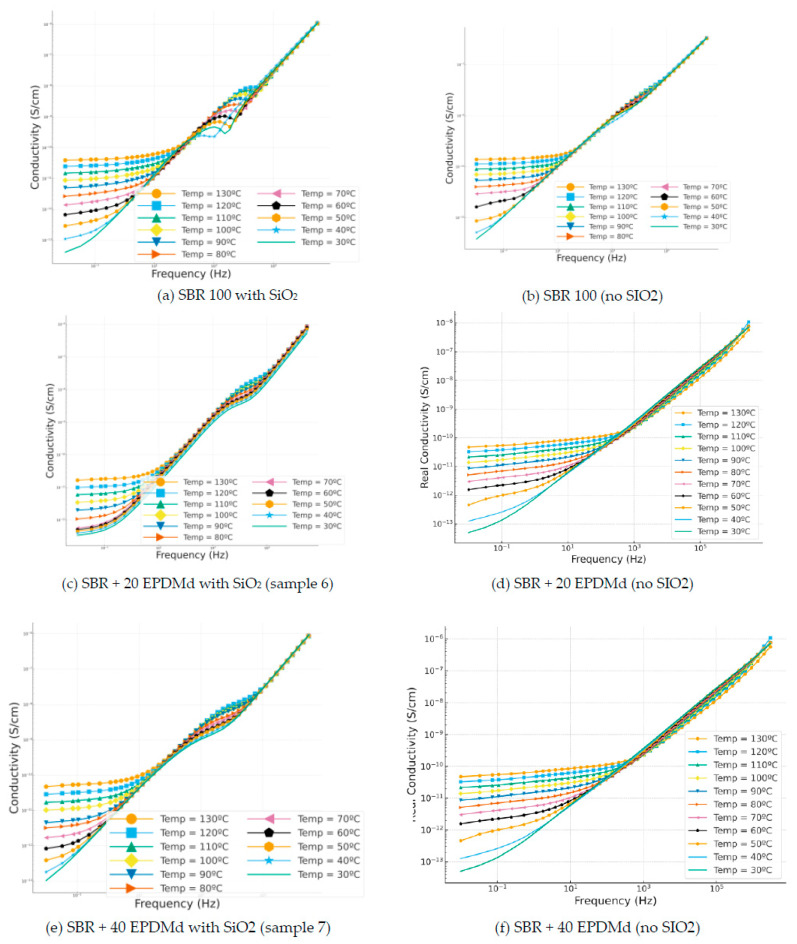
Conductivity (σ) versus frequency (Hz) in temperature dependence (°C) of Styrene Butadiene Rubber (SBR) and SBR-EPDMd composites, with SiO_2_ and no SiO_2_ addition: (**a**) SBR 100 with SiO_2_; (**b**) SBR 100; (**c**) SBR 100 + 20EPDMd with SiO_2_; (**d**) SBR + 20%EPDMd; (**e**) SBR 100 + 20EPDMd with SiO_2_; (**f**) SBR + 40%EPDMd.

In Figure 9, we can see a decrease in DC and AC conductivity with the presence of SiO_2_. Therefore, we have more insulating and resistive samples, making them more suitable as electrical insulation. The conductivity in polymeric materials is divided into two regimes, the AC regime (σ_ac_) and the DC regime (σ_dc_), which configure the conductivity in these types of materials and which are related by the following expression [3,34]:

$$\sigma_{\mathrm{ac}}\;=\;\sigma_{\mathrm{dc}}\;+\;A\cdot \omega^{s};$$

where A and s are parameters related to the temperature and additives used, and where w is the frequency (w = 2·π·f). In addition, σ_ac_ can be expressed as follows:

$$\sigma_{\mathrm{ac}}\;=\;\varepsilon_{0}\;\cdot \;\omega \;\cdot \;\varepsilon \prime \prime$$

Broadly speaking, we see how the DC conductivity is affected by temperature [35]; it is therefore a variable that is conditioned by temperature (30–130 °C). This is not so for the AC conductivity, which we see is independent of temperature and which presents a very similar behavior with temperature; it is but a variable (AC) dependent on frequency (Hz): increases in frequency translate into increases in conductivity. The materials that produce these compounds in all cases are electrical insulators, since we find ourselves in conductivities lower than 10^−10^ S/cm. Still, it would be necessary to analyze in more depth their industrial application as industrial insulators. In Figure 8c, we observe how the compound behaves when the DC conductivity is lower, below 10^−11^ S/cm, and it is the most conductive of the analyzed compounds. On the other hand, the temperature clearly conditions the DC conductivity; the higher the temperature, the higher the observed conductivity [36,37].

## 4. Conclusions

The main conclusions of the present research are that the SBR-EPDMd-SiO_2_ compound presents adequate structural, thermal, and dielectric characteristics. That is, structurally, large agglomerations of silica additive do not occur; on the other hand, at a thermal level, the peak shown in the DTGA, which indicates the degree of material degradation as a function of time, produces a relevant change (significant reduction) with 40% silica in the compound. This can be explained because silica in this compound already has an important presence, and this additive (SiO_2_) contributes decisively to this reduction in the thermal peak in this thermal behavior.

On the other hand, at the dielectric level, the material with the best characteristics as a dielectric insulator is the SBR-20EPDMd-SiO_2_ compound, since it presents conductivities below 10^−11^ S/cm. On the other hand, the relaxation peaks of the MWS relaxations that have been caused by silica are observed very clearly in the compounds with SiO_2_; these relaxations are not observed in the SBR-EPDMd composite without silica. On the other hand, at low frequencies, beta-type dielectric relaxations are observed in all cases, due to the movements of side groups of the main chain of the compounds. From a dielectric point of view, the insulator has the following conductivity values in S/cm: silicone rubber, 10^−14^ S/cm; EPDM rubber, 10^−13^ S/cm. Regarding permittivity, we can find standard values of the real permittivity. Relative permittivity ε′_r_ (real part): silicone rubber, 2.8–3.3; EPDM rubber, 2.3–2.8. The composites measure conductivities higher than the mentioned values for rubber but rise over 3 in the silicon composites, so, for capacitive applications, these type of rubber–silicon composites could be useful.

In future work, it would be necessary to analyze the abrasive properties of these analyzed compounds to determine whether they can be applied to electrical or mechanical insulation, or be reused in applications like the original ones of the initial EPDM material.

## Figures and Tables

**Table 1:** SBR-based rubber compounds containing devulcanized EPDM and their abbreviations (values in phr).

Abbreviation	SBR	EPDMd	SiO_2_	CB	ZnO	StA	TBBS	TMTD	S
SBR SiO_2_	100	0	30	30	5	3	1	0.25	2
SBR 20EPDMd SiO_2_	100	20	30	30	5	3	1	0.25	2
SBR 40EPDMd SiO_2_	100	40	30	30	5	3	1	0.25	2

## Data Availability

The original contributions presented in this study are included in the article. Further inquiries can be directed to the corresponding author.

## References

[1] Sutanto P., Picchioni F., Janssen L.P.B.M., Dijkhuis K.A.J., Dierkes W.K., Noordermeer J.W.M. (2006). State of the Art: Recycling of EPDM Rubber Vulcanizates Sutanto. Int. Polym. Process..

[2] Zhang G., Zhou X., Liang K., Guo B., Li X., Wang Z., Zhang L. (2019). Mechanically Robust and Recyclable EPDM Rubber Composites by a Green Cross-Linking Strategy. ACS Sustain. Chem. Eng..

[3] Marín-Genescà M., Mujal-Rosas R., García-Amorós J., Mudarra M., Ramis Juan X., Colom Fajula X. (2021). Study Analysis of Thermal, Dielectric, and Functional Characteristics of an Ethylene Polyethylene Diene Monomer Blended with End-of-Life Tire Microparticles Amounts. Polymers.

[4] Renukappa N.M., Siddaramaiah, Samuel S., Devaraju R.D. (2009). Dielectric properties of carbon black: SBR composites. J. Mater. Sci. Mater. Electron..

[5] Berki P., Göbl R., Karger-Kocsis J. (2017). Structure and properties of styrene-butadiene rubber (SBR) with pyrolytic and industrial carbon black. Polym. Test..

[6] Wiśniewska P., Wang S., Formela K. (2022). Waste tire rubber devulcanization technologies: State-of-the-art, limitations and future perspectives. Waste Manag..

[7] Markl E., Lackner M. (2020). Devulcanization Technologies for Recycling of Tire-Derived Rubber: A Review. Materials.

[8] Colom X., Marín-Genescà M., Mujal R., Formela K., Cañavate J. (2018). Structural and physico-mechanical properties of natural rubber/GTR composites devulcanized by microwaves: Influence of GTR source and irradiation time. J. Compos. Mater..

[9] Sabzekar M., Pourafshari Chenar M., Mortazavi S.M., Kariminejad M., Asadi S., Zohuri G. (2015). Influence of process variables on chemical devulcanization of sulfur-cured natural rubber. Polym. Degrad. Stab..

[10] Guo L., Wang C., Lv D., Ren D., Zhai T., Sun C., Liu H. (2021). Rubber reclamation with high bond-breaking selectivity using a low-temperature mechano-chemical devulcanization method. J. Clean. Prod..

[11] Ismail H., Freakley P.K., Sutherland I., Sheng E. (1995). Effects of multifunctional additive on mechanical properties of silica filled natural rubber compound. Eur. Polym. J..

[12] Sisanth K.S., Thomas M.G., Abraham J., Thomas S., Sabu T., Hanna J.M. (2017). 1—General introduction to rubber compounding. Progress in Rubber Nanocomposites.

[13] Carr S.W., Courtney L., Sullivan A.C. (1997). Effects of Molecular Organic Additives on Formation and Properties of Organosilicate and Silica Xerogels Correlated to Structural Properties of the Additive. Chem. Mater..

[14] Zedler Ł., Colom X., Cañavate J., Formela K. (2021). GTR/NBR/Silica Composites Performance Properties as a Function of Curing System: Sulfur versus Peroxides. Materials.

[15] Kyriazis A., Charif S., Rager K., Dietzel A., Sinapius M. (2023). A parametric model for the impedance spectra of polymer composites. Sensors.

[16] Heinrich G. (2021). Rubber Chemistry and Technology.

[17] Marín-Genescà M., García-Amorós J., Mujal-Rosas R., Massagués L., Colom X. (2020). Study and Characterization of the Dielectric Behavior of Low Linear Density Polyethylene Composites Mixed with Ground Tire Rubber Particles. Polymers.

[18] Vogelwaid J., Hampel F., Bayer M., Walz M., Kutuzova L., Lorenz G., Kandelbauer A., Jacob T. (2024). In situ monitoring of the curing of highly filled epoxy molding compounds. Polymers.

[19] Colom X., Marín M., Saeb M.R., Formela K., Cañavate J. (2024). Recycling devulcanized EPDM to improve engineering properties of SBR rubber compounds. Resour. Conserv. Recycl. Adv..

[20] Marín-Genescà M., García-Amorós J., Mudarra M., Massagués Vidal L., Cañavate J., Colom X. (2023). Insights into the structural and dielectric behavior of composites produced from EPDM waste processed through a devulcanization method and SBR. ACS Omega.

[21] Perez L.D., Lopez B.L. (2012). Thermal characterization of SBR/NBR blends reinforced with a mesoporous silica. J. Appl. Polym. Sci..

[22] Goyanes S., Lopez C.C., Rubiolo G.H., Quasso F., Marzocca A.J. (2008). Thermal properties in cured natural rubber/styrene butadiene rubber blends. Eur. Polym. J..

[23] Zhang Y., Zhang Q., Liu Q., Cheng H., Frost R.L. (2014). Thermal stability of styrene butadiene rubber (SBR) composites filled with kaolinite/silica hybrid filler. J. Therm. Anal. Calorim..

[24] Tian M., Qu C., Feng Y., Zhang L. (2003). Structure and properties of fibrillar silicate/SBR composites by direct blend process. J. Mater. Sci..

[25] Zhao S., Zhang X., Jin G. (2003). Influence of molecular structure of star S-SBR on its properties. J. Appl. Polym. Sci..

[26] Sadhu S., Bhowmick A.K. (2005). Morphology study of rubber based nanocomposites by transmission electron microscopy and atomic force microscopy. J. Mater. Sci..

[27] Qiu J., Gu Q., Sha Y., Huang Y., Zhang M., Luo Z. (2022). Preparation and application of dielectric polymers with high permittivity and low energy loss: A mini review. Appl. Polym. Sci..

[28] Ogitani S., Bidstrup-Allen S.A., Kohl P.A. (2000). Factors influencing the permittivity of polymer/ceramic composites for embedded capacitor. IEEE Trans. Adv. Packag..

[29] Marín-Genescà M., García-Amorós J., Mujal-Rosas R., Massagués Vidal L., Colom Fajula X. (2020). Application Properties Analysis as a Dielectric Capacitor of End-of-Life Tire-Reinforced HDPE. Polymers.

[30] Chaudhury S., Sinha S.K. (2019). Chapter 12—Carbon Nanotube and Nanowires for Future Semiconductor Devices Applications. Nanoelectronics; Advanced Nanomaterials.

[31] Hanna F.F., Yehia A.A., Abou-Bakr A.-F. (1973). Dielectric Properties of Styrene—Butadiene Rubber/Silicon Dioxide Mixtures. Brit. Poly. J..

[32] Mujal-Rosas R., Marin-Genesca M., Orrit-Prat J., Rahhali A., Colom-Fajula X. (2012). Dielectric, mechanical, and thermal characterization of high-density polyethylene composites with ground tire rubber. J. Thermoplast. Compos. Mater..

[33] Mujal-Rosas R., Orrit-Prat J., Ramis-Juan X., Marin-Genesca M., Rahhali A. (2013). Dielectric, Thermal, and Mechanical Properties of Acrylonitrile Butadiene Styrene Reinforced with Used Tires. Adv. Polym. Technol..

[34] Orrit-Prat J., Mujal-Rosas R., Rahhali A., Marín-Genescà M., Colom-Fajula X., Belana-Punseti J. (2010). Dielectric and mechanical characterization of PVC composites with ground tire rubber. J. Compos. Mater..

[35] Marín-Genescà M., García-Amorós J., Mujal-Rosas R., Salueña Berna X., Massagués Vidal L. (2019). Comparison of Mechanical and Electrical Characteristics of Various Polymers Blended with Ground Tire Rubber (GTR) and Applications. Appl. Sci..

[36] Marín-Genescà M., Mujal Rosas R., García Amorós J., Massagues Vidal L., Colom Fajula X. (2021). Influence of Tire Rubber Particles Addition in Different Branching Degrees Polyethylene Matrix Composites on Physical and Structural Behavior. Polymers.

[37] Doma A.S., El-Khatib A.M., Abu-Rayan A.E., Wazeer W., Abbas M.I. (2022). A study on some physical properties of conductive Pb/NR composites. J. Rubber Res..

